# Hepatic Cyst Radioiodine Uptake on Whole-Body Scan in Differentiated Thyroid Cancer: Implications for Lesion Characterization and Misdiagnosis Avoidance

**DOI:** 10.7759/cureus.82789

**Published:** 2025-04-22

**Authors:** Raghad Al-houwari, Serin Moghrabi, Mohammad Abu Shattal, Akram Al-Ibraheem

**Affiliations:** 1 Nuclear Medicine, King Hussein Cancer Center, Amman, JOR; 2 Diagnostic Radiology, King Hussein Cancer Center, Amman, JOR

**Keywords:** havm, liver cyst, pitfalls, rai, thyroid cancer

## Abstract

Radioactive iodine (RAI) imaging constitutes a fundamental diagnostic tool in post-operative thyroid cancer management. A diverse spectrum of uptake patterns, encompassing both physiological and pathological entities, has been reported in patients undergoing RAI studies. Particularly, a variety of non-thyroidal conditions can present with unusual patterns of RAI uptake in the liver, complicating diagnosis. This case report presents a 51-year-old female with papillary thyroid cancer who underwent a whole-body RAI scan. The study revealed an atypical pattern of increased radiotracer concentration within the liver for which subsequent radiological investigations led to the diagnosis of two liver lesions, one was a simple hepatic cyst and another was most likely to be a hepatic arteriovenous malformation (HAVM) both located in segment VII based on abdominal magnetic resonance imaging (MRI) findings, an unexpected finding that could be mistaken for other differential diagnoses, including metastatic differentiated thyroid cancer (DTC), granulomas, abscesses, hydatid cysts, biliary tract dilatation, and other benign non-thyroidal neoplasia. Recognizing these diverse entities is essential for the accurate interpretation of RAI scans and for avoiding misdiagnosis. This finding may help clinicians avoid misdiagnosing such ancillary observations as metastases, thereby improving diagnostic accuracy in thyroid cancer management.

## Introduction

Differentiated thyroid carcinoma (DTC), comprising papillary, follicular, and oncocytic thyroid cancers, represents the most common type of thyroid malignancy, making up about 85%-90% of all cases [1]. The incidence of DTC has significantly increased over the past few decades [1]. This increase is primarily attributed to the widespread use of sensitive diagnostic imaging techniques, such as high-resolution ultrasound and fine-needle aspiration biopsy, which have led to the early detection of smaller, often indolent tumors that might have previously gone unnoticed [2].

Despite this surge in incidence, the overall prognosis for DTC remains excellent, with a 10-year survival rate exceeding 90% [1]. This favorable outcome may be largely attributable to the generally slow-growing nature of these tumors and the effectiveness of contemporary treatment strategies, including surgical resection such as total or hemi-thyroidectomy, radioactive iodine (RAI) therapy, and tyrosine kinase inhibitors [1]. The use of RAI in treating DTC relies on the fact that some thyroid cancer cells continue to express the sodium-iodide symporter (NIS) [3]. However, NIS expression is not exclusive to thyroid cells, leading to potentially false-positive results on RAI whole-body scans [3]. Therefore, unexpected iodine uptake findings should be interpreted considering the patient's medical history, thyroglobulin levels, and tumor histopathology, and corroborated with cross-sectional imaging studies [3].

According to the categorization established by Brucker-Davis et al., false-positive results from whole-body 131I scans can be broadly categorized into four distinct groups, namely the excretion of iodine in bodily fluids, infectious or inflammatory processes, the presence of cysts or transudates, and neoplasms originating from non-thyroidal tissues [4]. Some of these coexisting pathological conditions can influence diagnostic interpretation and clinical outcomes, underscoring the importance of accurate characterization [5]. Over the past several decades, numerous studies have underscored the valuable potential of non-thyroidal tissues to accumulate RAI. These investigations have revealed that certain extrathyroidal tissues may possess the capacity to take up RAI through various mechanisms, some of which remain inadequately understood [3,6]. This phenomenon suggests a complexity in iodine metabolism and transport that extends beyond the known thyroid physiology, prompting further exploration into the underlying pathways and implications for both diagnostic and therapeutic applications in nuclear medicine.

In this report, we present a unique case of RAI uptake in a simple liver cyst near a small non-RAI-avid hepatic arteriovenous malformation (HAVM). This case underscores the importance of careful interpretation of RAI uptake, as it can lead to unexpected findings that may not be directly related to thyroid tissue or malignancy. This case highlights the need for a multidisciplinary approach in the management of DTC, especially when confronted with atypical findings, to ensure accurate diagnosis and appropriate treatment.

## Case presentation

A medically and surgically free 51-year-old female patient with a history of papillary thyroid cancer, oncocytic variant, since 2014, underwent total thyroidectomy in November 2014, and after pathological analysis, the TNM stage was (pT3Nx). Accordingly, the patient was considered to have an intermediate risk of recurrence and received her first dose of RAI therapy, 4440 megabecquerel (MBq), in December 2014. Subsequent whole-body RAI scan indicated an RAI uptake in the remnant thyroid tissue, which completely resolved on the follow-up scan six months later.

In April 2022, the patient was referred to a tertiary center due to a pathologically abnormal left level VI lymph node detected on a neck ultrasound (US), measuring approximately 0.7 cm during a routine check-up. Her thyroid-stimulating hormone (TSH) level was 30 µIU/mL, and her stimulated thyroglobulin (sTg) level was 12.3 ng/mL, with normal anti-thyroglobulin antibody levels. Fine-needle aspiration (FNA) of the lymph node confirmed papillary thyroid carcinoma. The multidisciplinary team (MDT) recommended a second dose of RAI therapy (4,625 MBq), which was administered in August 2022. At that time, her TSH and sTg levels were 57 µIU/mL and 50 ng/mL, respectively.

**Figure 1:**
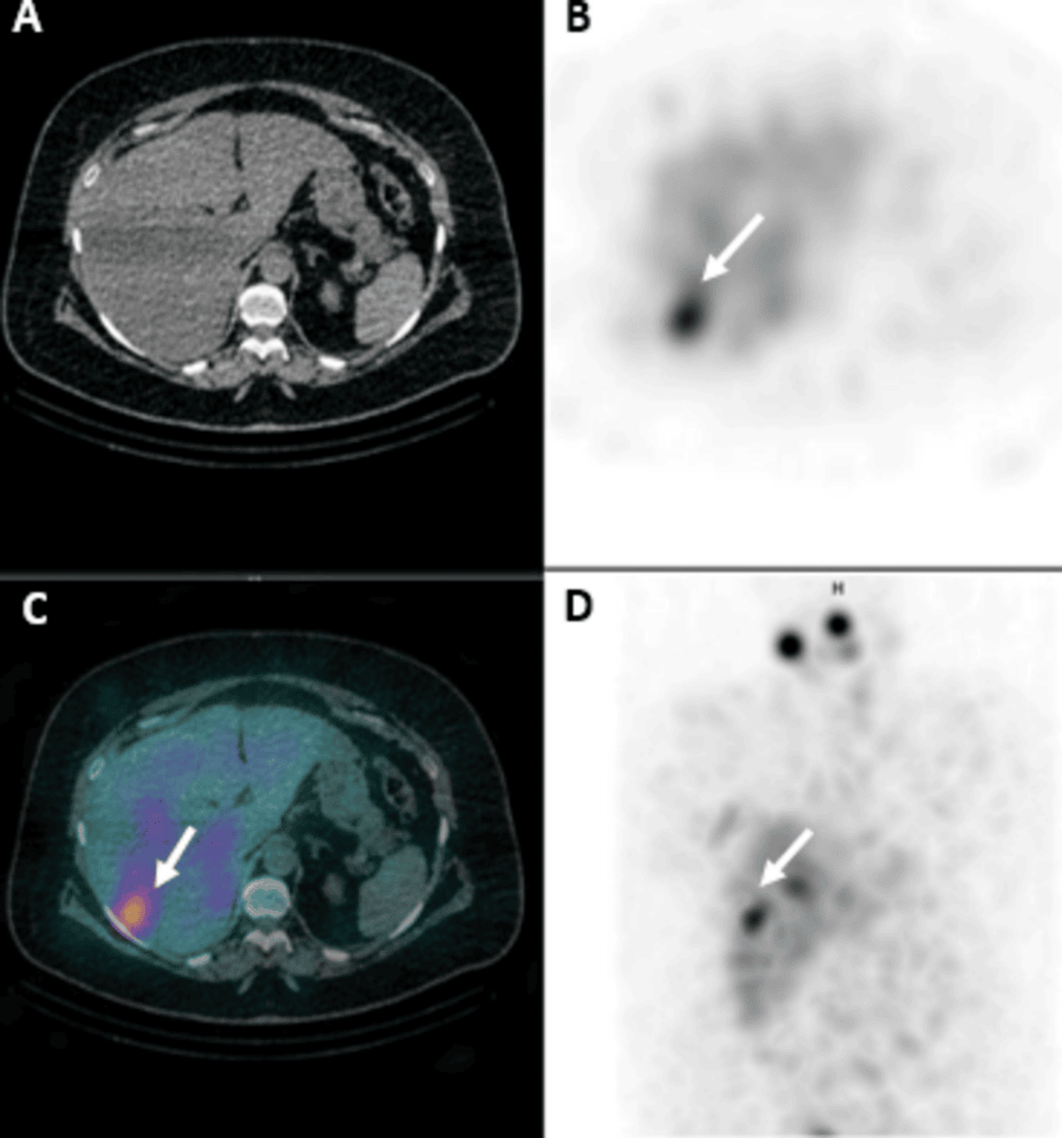
Post-ablation whole-body RAI SPECT/CT (second RAI dose). Post-ablation whole-body RAI SPECT/CT done one week after the second RAI dose shows RAI uptake (white arrow) within hepatic segment VII seen in SPECT, fused, and MIP images (B, C, and D, respectively), not corresponding to definite CT changes (A) with non-specific RAI activity in the remaining liver. RAI, radioactive iodine; SPECT/CT, single photon emission computed tomography/computed tomography; MIP, maximum intensity projection

Post-ablation whole-body RAI single photon emission computed tomography/computed tomography (SPECT/CT) scan one week later revealed several small lymph nodes not showing RAI uptake, including a prominent left level VI lymph node measuring approximately 0.8 cm. Additionally, there was a concerning focus of increased RAI uptake within hepatic segment VII, which did not correspond to definite changes on non-contrast CT, with non-specific, diffusely minimal RAI activity in the remainder of the liver (Figure 1). Given the elevated sTg levels, metastatic involvement could not be excluded, and further magnetic resonance imaging (MRI) evaluation was advised.

**Figure 2:**
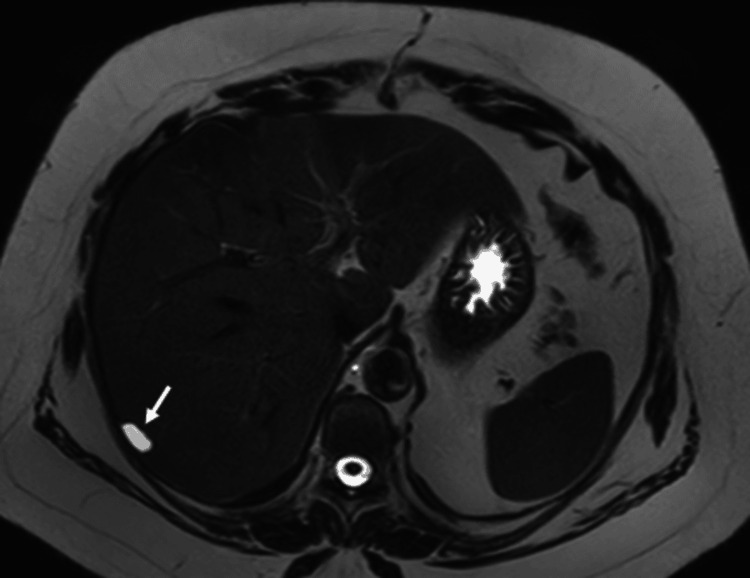
Abdominal MRI (liver cyst). Short-TI inversion recovery (STIR) abdominal MRI scan shows a hypervascular lesion in segment VII of the right liver lobe, measuring about 1.6 cm, retaining IV contrast (white arrow).

**Figure 3:**
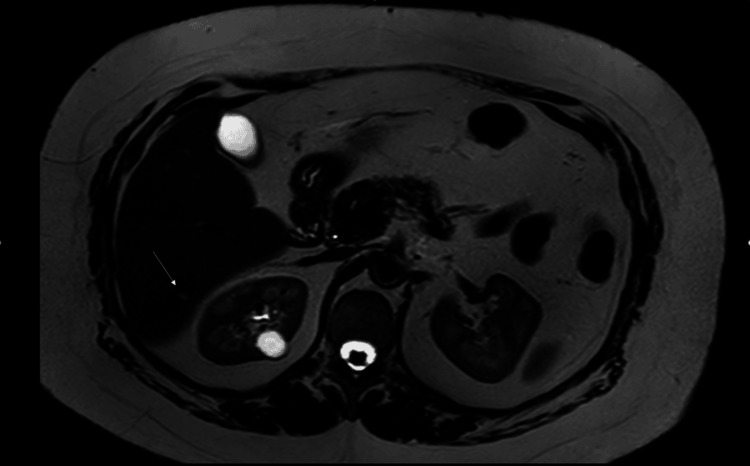
Abdominal MRI (HAVM). STIR abdominal MRI shows an early enhancing lesion in segment VII of the right liver lobe (white arrow), measuring about 1.6 cm, the appearance of which is suggestive of a small arteriovenous malformation. HAVM, hepatic arteriovenous malformation; STIR, short-TI inversion recovery

In January 2023, an abdominal MRI revealed a non-enhancing small subcapsular liver cyst in segment VII of the right liver lobe with a nearby sub-centimetric hypervascular early-enhancing lesion retaining intravenous contrast in the delayed phase. This appearance was suggestive of a benign cyst with associated small HAVM. No intervention was taken to treat the cyst or the HAVM, as they were small, asymptomatic, and posed a low risk of rupture and bleeding, respectively (Figures 2, 3).

**Figure 4:**
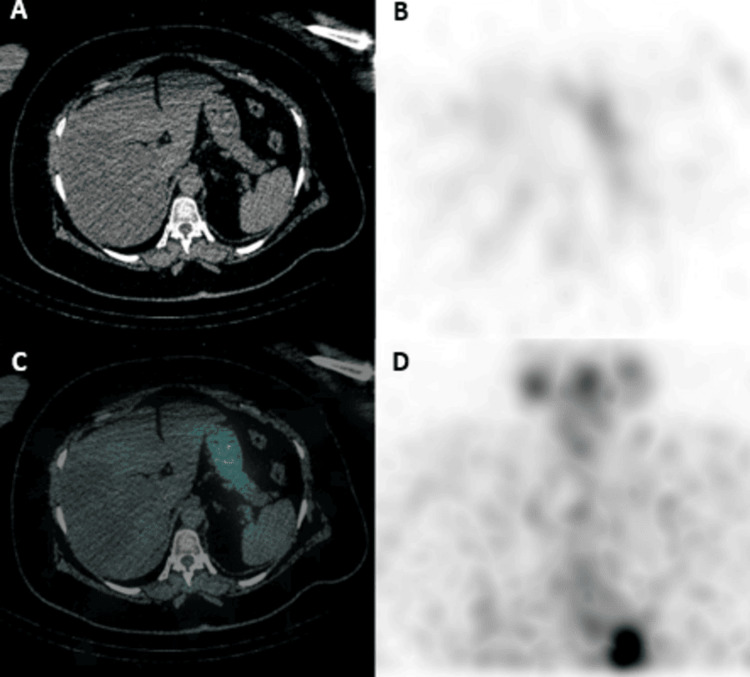
Diagnostic whole-body RAI SPECT/CT scan (second RAI dose). Diagnostic whole-body RAI SPECT/CT scan done six months after the second RAI dose does not currently show RAI uptake within the hepatic segment VII in SPECT, fused, and MIP images (B, C, and D, respectively). RAI, radioactive iodine; SPECT/CT, single photon emission computed tomography/computed tomography; MIP, maximum intensity projection

A six-month workup included a diagnostic whole-body RAI SPECT/CT scan with a dose of 5 mCi in February 2023, which showed slight size progression of the lymph nodes, which still did not show RAI uptake, and the known focal area of increased RAI uptake within the liver (segment VII) was not appreciated in this scan (Figure 4). A neck US also showed an increase in the size of the known left deep cervical lymph node at level VI, now measuring 1.0 cm compared to the previous 0.7 cm in the short axis. At this time, the patient’s TSH and sTg levels were 39.6 µIU/mL and 65 ng/mL, respectively.

A fluorodeoxyglucose positron emission tomography/CT (FDG PET/CT) scan performed subsequently revealed intense FDG uptake in the cervical lymph nodes in left levels IVb and VIb, with a maximum standardized uptake value (SUVmax) of 25.0. However, there was a physiologically homogenous FDG uptake in the liver with no appreciation of the known cyst or HAVM on the PET part of the study. The MDT planned a central lymph node and left modified radical neck dissection in April 2023. Histopathology confirmed metastatic involvement by papillary thyroid cancer in one out of four left para-tracheal lymph nodes and four out of forty-three left neck lymph nodes.

**Figure 5:**
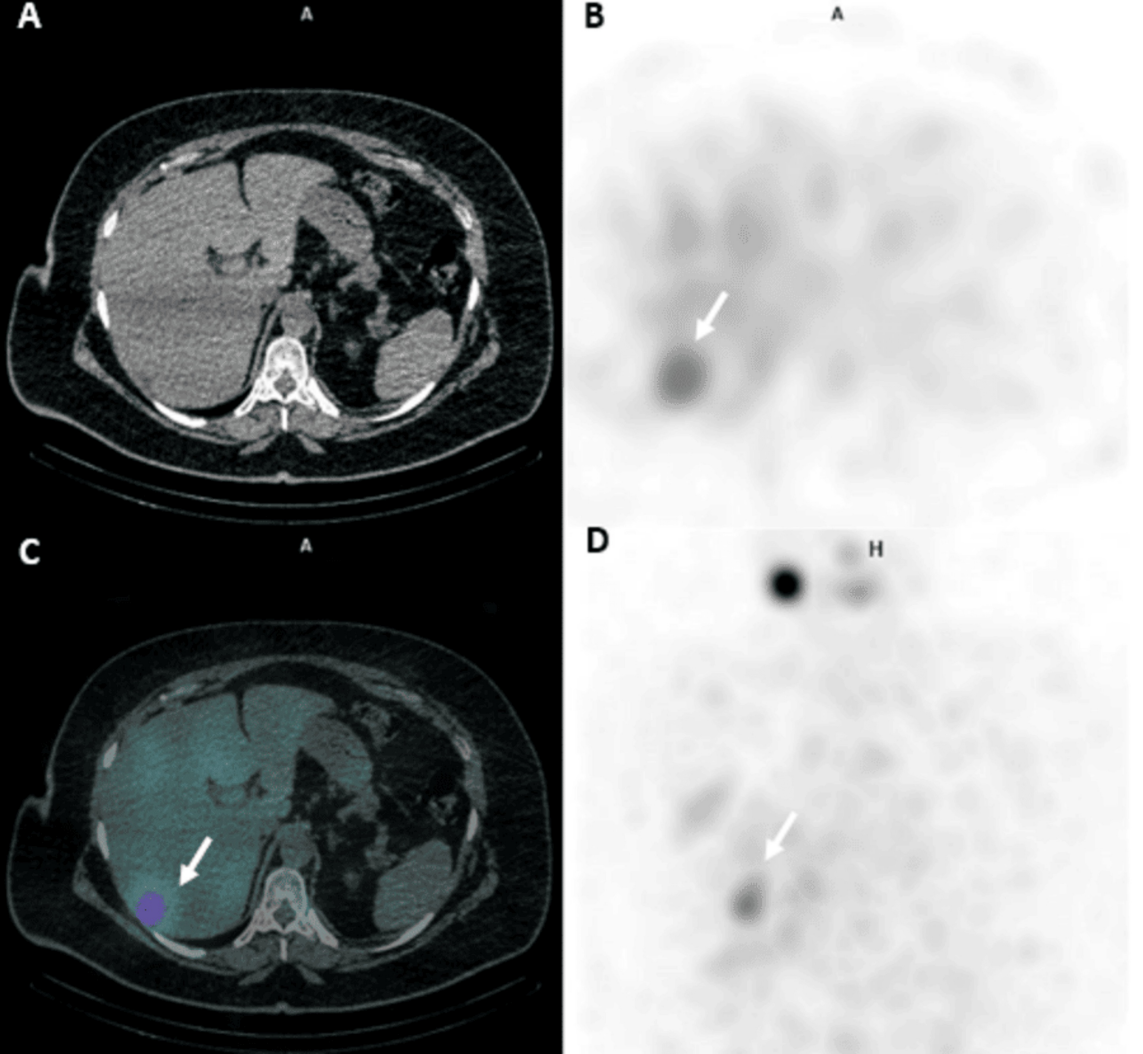
Post-ablation whole-body RAI SPECT/CT done (third RAI dose). Post-ablation whole-body RAI SPECT/CT done one week after the third RAI dose shows reappearance of the RAI uptake within hepatic lesion in segment VII (white arrow) demonstrated on SPECT, fused, and MIP images (B, C, and D respectively). RAI, radioactive iodine; SPECT/CT, single photon emission computed tomography/computed tomography; MIP, maximum intensity projection

**Figure 6:**
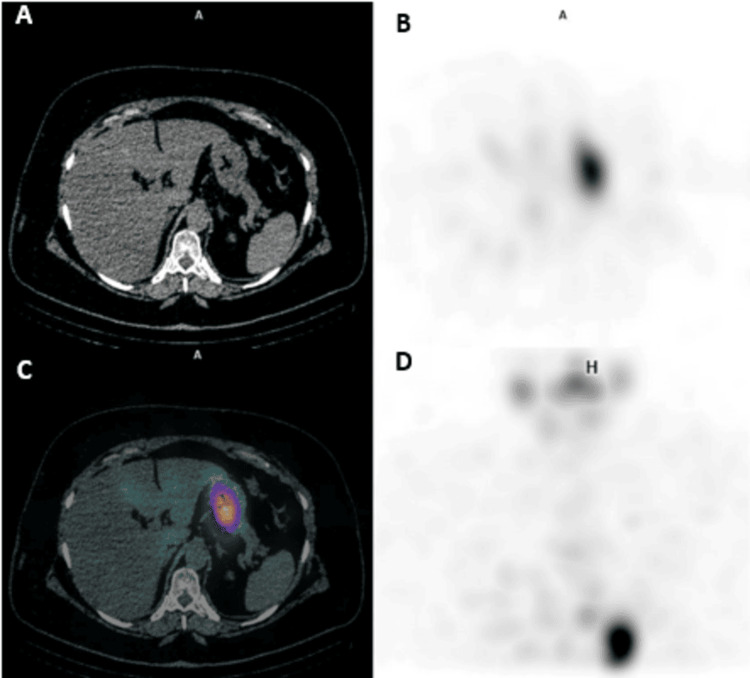
Diagnostic whole-body RAI SPECT/CT scan (third RAI dose). Diagnostic whole-body RAI SPECT/CT scan done six months after the third RAI dose shows complete resolution of the RAI uptake within hepatic segment VII in SPECT, fused, and MIP images (B, C, and D respectively). RAI, radioactive iodine; SPECT/CT, single photon emission computed tomography/computed tomography; MIP, maximum intensity projection

Subsequent lab results in May 2023 indicated TSH and sTg levels of 95 µIU/mL and 3 ng/mL, respectively. A third dose of RAI (4,625 MBq) was administered in June 2023. Post-ablation whole-body RAI SPECT/CT scans showed no convincing evidence of RAI lesions in the neck but revealed the reappearance of the RAI uptake in the hepatic lesion within segment VII (Figure 5), likely corresponding to the subcapsular benign cyst seen on MRI, which again became unappreciated on the diagnostic whole-body RAI SPECT/CT scan performed six months later, in December 2023 (Figure 6).

## Discussion

Following thyroidectomy in patients diagnosed with DTC, RAI is often applied as a supplementary therapeutic intervention that, in some patients, aims to achieve an ablative function of remaining thyroid tissue [6]. However, false-positive RAI uptake can occur, leading to misinterpretation of imaging results [6]. These false positives may arise from various non-thyroidal tissues and conditions exhibiting RAI uptake, such as inflammation, infection, benign and malignant neoplasms, physiological uptake in salivary glands, thymus, gastrointestinal tract, and even surgical scars [6].

In our case, a 51-year-old female with papillary thyroid cancer exhibited increased RAI uptake in the liver, raising concerns for metastasis due to elevated sTg levels. Subsequent MRI revealed a sub-capsular hepatic cyst and an adjacent hypervascular lesion consistent with an HAVM.

The diffuse RAI uptake in the liver is frequently reported in RAI whole-body scans, occurring in 40%-94% of patients, but the exact mechanism remains unclear [3]. One proposed explanation is the hepatic metabolism of organified RAI. Intrahepatic bile duct NIS expression is another hypothesized mechanism, with some reports indicating a correlation between hepatic uptake and the RAI dose [3]. In addition, a higher administered dose of RAI contributes to the increased physiological hepatic uptake seen on whole-body RAI scans, which may explain the lower RAI uptake on diagnostic scans as opposed to the notable uptake on post-therapeutic ones [3].

The importance of accurately diagnosing localized liver abnormalities cannot be overstated, as it directly impacts the efficacy of subsequent treatment protocols. Particularly, focal liver lesions, which can comprise a diverse array of benign and malignant conditions, are commonly detected by radiologists [7].

Hepatic cysts are a relatively common finding in radiological examinations as an incidental finding, affecting approximately 2%-7% of the general population, with a higher propensity in females [8]. Simple hepatic cysts cannot generally absorb RAI, as the uptake of RAI is predominantly linked to thyroid tissue and specific neoplastic entities that express NIS, which are absent in hepatic cysts. However, this case is among the few reported RAI-avid hepatic cysts in the literature, in which RAI was unexpectedly detected in a focal liver lesion in a DTC patient, raising the possibility of a potential metastatic process. Okuyama et al. reported the first case of RAI uptake in a benign hepatic cyst, which was presumably ascribed to a gradual exchange of water and chemical constituents between the cyst and its surrounding milieu via a passive or at certain times, active mechanism and since then, only a few more studies reported similar incidental findings [9-12]. RAI avidity in hepatic cysts is a rare occurrence. A review of the literature reveals only six previously published cases, including the present case, to our knowledge. This limited number highlights the unusual nature of this finding and emphasizes the importance of considering this possibility in the differential diagnosis of RAI-avid lesions in the abdomen.

Arteriovenous malformations can occur in the liver and are characterized by abnormal connections between the hepatic arteries and portal veins [13]. The literature offers limited case reports denoting the infrequent presentation of HAVMs across diverse age groups, from fetal to adult life [13,14]. While a subset of HAVMs may be linked to hereditary hemorrhagic telangiectasia, the etiology of many HAVMs remains unclear [15]. Diagnosis is typically made through imaging studies, such as ultrasound, contrast-enhanced CT, and MRI. US may reveal a hypervascular lesion with turbulent blood flow, and MRI may reveal brisk enhancement or, as in our case, early arterial enhancement and prolonged contrast retention in the delayed phase [16]. Nevertheless, to date, the literature lacks definitive evidence establishing a direct correlation between RAI uptake and the presence of HAVMs.

Other potential causes of hepatic RAI uptake include liver metastases, which have a frequency of 0.5% in patients with DTC [17]. However, liver metastases are better characterized by contrast-enhanced abdominal MRI, which can help distinguish between benign lesions and metastatic involvement [18]. In our case, MRI effectively excluded the presence of liver metastases. Also, inflammatory and infectious conditions show RAI liver uptake, which usually shows regression upon antibiotic treatment [19]. Hepatic hemangioma shows increased RAI uptake but can be identified by contrast-enhanced CT [20].

This understanding is crucial for interpreting imaging results in patients undergoing treatment for thyroid conditions since this case report represents an important example illustrating two closely related incidental hepatic findings with differing responses to radioactive iodine, one being receptive and the other not, and thus enhancing our understanding of similar uptake patterns as well as the potential to inspire further noticing of similar occurrences, marking a noteworthy addition to the medical literature.

## Conclusions

This case report presents a novel observation as the first documented case of an RAI-avid hepatic cyst occurring adjacent to a non-RAI-avid HAVM. This unique presentation adds significantly to the limited literature on this rare phenomenon, with only five previously published cases about RAI uptake in hepatic cysts. It also highlights the importance of considering this diagnostic possibility in patients presenting with unusual patterns of RAI uptake, which can be misinterpreted as metastatic disease.
